# Time Course of the Soleus M Response and H Reflex after Lidocaine Tibial Nerve Block in the Rat

**DOI:** 10.1155/2013/912716

**Published:** 2013-08-07

**Authors:** Kévin Buffenoir, Philippe Decq, Chantal Pérot

**Affiliations:** ^1^Université de Technologie de Compiègne, CNRS UMR 7338 Biomécanique et Bioingénierie, BP 20529, 60205 Compiègne, France; ^2^Service de Neurotraumatologie, CHU Hôtel Dieu, 1 Place Alexis Ricordeau, 44093 Nantes, France; ^3^Equipe Biomécanique et Système Nerveux. LBM ENSAM ParisTech, Faculté de Médecine PARIS 12, Assistance Publique—Hôpitaux de Paris, Service de Neurochirurgie, Hôpital Henri Mondor, 51 avenue du Maréchal de Lattre de Tassigny, 94010 Créteil, France

## Abstract

*Aims*. In spastic subjects, lidocaine is often used to induce a block predictive of the result provided by subsequent surgery. Lidocaine has been demonstrated to inhibit the Hoffmann (H) reflex to a greater extent than the direct motor (M) response induced by electrical stimulation, but the timecourse of these responses has not been investigated. *Methods*. An animal (rat) model of the effects of lidocaine on M and H responses was therefore developed to assess this time course. M and H responses were recorded in 18 adult rats before and after application of lidocaine to the sciatic nerve. *Results*. Two to five minutes after lidocaine injection, M responses were markedly reduced (mean reduction of 44%) and H reflexes were completely abolished. Changes were observed more rapidly for the H reflex. The effects of lidocaine then persisted for 100 minutes. The effect of lidocaine was therefore more prolonged on the H reflex than on the M response. *Conclusion*. This study confirms that lidocaine blocks not only alpha motoneurons but also Ia afferent fibres responsible for the H reflex. The authors describe, for the first time, the detailed time course of the effect of lidocaine on direct or reflex activation of motoneurons in the rat.

## 1. Introduction

Lidocaine peripheral nerve block, described by Tardieu and Harriga in 1964 [1], is used in the evaluation of patients with spastic equinus foot as a diagnostic and therapeutic test before proposing selective tibial neurotomy [2–5]. A recent study of the effects of lidocaine blocks on H and M responses in man showed more intense inhibition of the H reflex than the M response, suggesting a predominant effect of lidocaine on Ia afferent fibres compared to alpha motoneurons [5]. Our laboratory has also developed a technique for the recording of H and M responses by surface electromyography in rats [6]. In order to study the time course of installation, maintenance, and abolition of the nerve block induced by lidocaine, we therefore considered using this rat model of electromyographic recording of M (direct activation of alpha motoneurons) and H responses (activation of Ia afferent fibres). Few authors [7–11] have studied the effects of lidocaine in rats, and these studies were based exclusively on clinical parameters. We are unaware of any published studies that reported modifications of reflex electrophysiological parameters in rats after application of lidocaine, which constitutes one of the original aspects of the present study.

## 2. Materials and Methods

**Figure 1:**
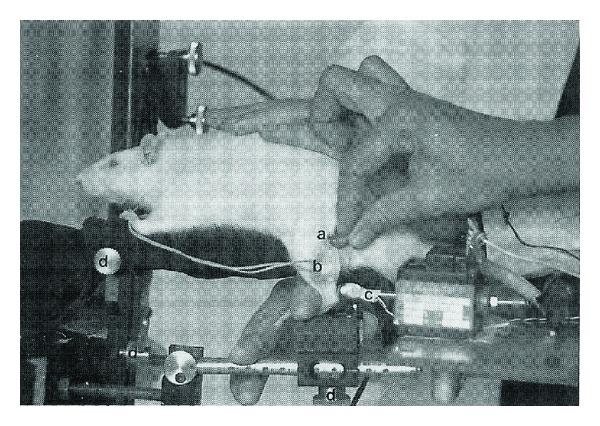
Illustration of the position of the rat during the “conscious” study, the positions of stimulation (a) and recording (b) electrodes, and the reflex hammer not used in this protocol (c). Various adjustments (d) can be made on the ergometer.

All experiments and rat housing conditions were approved by the French Ministry of Agriculture and Forestry (authorisation 04910). Eighteen adult Wistar rats (12 weeks old, body weight: 330 ± 30 g) were used for this study. H and M responses of the right plantar extensors were initially recorded in conscious rats according to a previously described technique [6]. The lateral surface of the paw was carefully shaved in the zone of the triceps surae, and two silver electrodes, 2 mm in diameter, chlorinated before use, were placed over the soleus muscle (situated more deeply in rats) and maintained by adhesive plaster. A reference electrode was placed in the animal's tail. After being fitted with these electrodes, the rat was placed in the prone position on a small platform allowing maintenance of the test paw in the vertical position, with the knee flexed to an angle of about 120° and the ankle flexed to an angle of 90° (Figure 1). Stimulation of the sciatic nerve was delivered via two silver electrodes moulded in a plastic support, fitted with a tip allowing the operator to maintain the electrodes applied along the sciatic nerve close to the knee. Electrical stimuli (Digitimer DS7A stimulator) were 0.2 ms in duration with an intensity ranging between 0 and 20 mA. Under conscious rat conditions, stimuli were triggered manually when the animal was perfectly calm and a trigger synchronously activated oscilloscope scanning (Tektronics SDR 3014) and data acquisition on the computer hard disk. During this experiment that lasted about 5 minutes, the animal and its paw were maintained manually by the operator, without using a restraint device, avoiding stress of the animal and allowing EMG recordings under conscious conditions. The EMG signals recorded were then amplified (Grass model CP511A amplifier) and filtered (band pass between 10 Hz and 1 KHZ). EMG data acquisition was performed by an analogue/digital conversion card using ACQ2000XP software developed in the laboratory. As classically performed in rats [6, 12], stimulation was increased to simultaneously obtain greater amplitude H and M responses. Fifteen to twenty stimuli were applied in this way, which subsequently allowed calculation of the mean M or H response from the ten largest responses obtained. Maximum M or H responses were characterized in terms of amplitude and latency. 

After recording electromyographic responses in conscious rats, the animals were anaesthetised by intraperitoneal injection of ketamine. Ketamine was chosen because it has been clearly described in the literature that this molecule does not induce any modification of H and M responses, unlike other molecules such as propofol and etomidate [13]. An initial injection of 150 mg/kg was administered and anaesthesia was then maintained by injections of 25 mg/kg that could be performed every 15 minutes, as necessary. All rats required a second injection, but only 3 rats required a third injection. 

**Figure 2:**
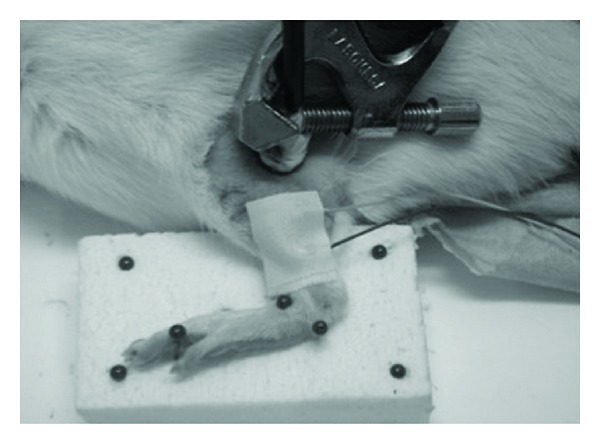
Illustration of the position of the rat's paw fixed to a support during the general anaesthesia study and the position of the stimulation and recording electrodes.

To record responses under anaesthesia, the rat was installed in the lateral supine position on an experimental table with the paw maintained by adhesive tape in the same standardized position (knee flexed to 120° and ankle flexed to 90°) as during the tests under conscious conditions. The protocol described previously was repeated with a few minor differences (Figure 2): (1) stimulation electrodes were attached to a support with a ball-and-socket joint allowing them to be applied just over the site of stimulation of the sciatic nerve, and to prevent any displacement during the experiment; (2) the frequency of stimulation depended on the phase of the experiment. The syringe containing lidocaine was attached to a support that could be adjusted in terms of height and laterally, and the 25 gauge needle was inserted between the greater trochanter and the ischial tuberosity until contact with the ischium. Once this position was achieved, the electrode support device was locked in position to avoid any subsequent displacement, including during the injection of lidocaine.

The experiment under anaesthesia began by testing the stimulation electrode positions and the intensity of stimulation that gave the highest amplitude M and H responses. The electrodes were then locked in position and 10 responses were recorded (interstimulus interval of 5 seconds).

After recording the reference responses under anaesthesia, percutaneous sciatic nerve block was performed by injection of 0.5 mL of 1% lidocaine. The first stimulus was delivered at the end of the injection and the following stimuli were delivered with an interstimulus interval of 30 seconds until the 10th minute then an interstimulus interval of 60 seconds until the 100th minute after injection. After the 100th minute, during the recovery phase, the interstimulus interval was 30 seconds and stimuli were delivered until complete recovery of H and M responses.

EMG activity was analysed by “Neuromecanik” software developed in the laboratory with Matlab (The MathWorks, Inc. Natick, MA 01760-2098, USA): the operator had to manually identify the start and end of M or H responses and the peak-to-peak amplitude and latencies of H and M responses were then calculated automatically. Finally, 4 rats were used to test the placebo effect. A complete H and M response recording protocol was performed before and after percutaneous injection of 0.5 cc of physiological saline in contact with the sciatic nerve according to the same methodology as that described earlier.

The rats were sacrificed at the end of the protocol. 

Statistical analyses were performed with StatView version 5.0 software (SAS Institute INC). A paired *t*-test was used for all comparisons with a limit of significance of 0.05.

## 3. Results

**Figure 3:**
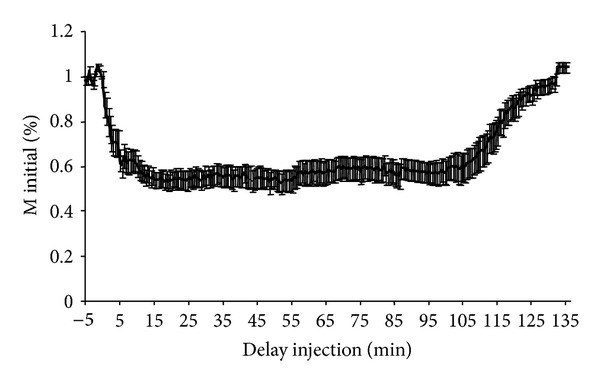
Mean time course of the amplitude of the M response expressed in relation to the baseline M response before and after application of lidocaine. Time (minutes) after application of lidocaine (performed at time 0) is shown on the *x*-axis. The mean amplitude of the M response of the 18 rats expressed in relation to the baseline M response (corresponding to the mean of 10 M responses studied before application of lidocaine) is shown on the *y*-axis. The interval shown on the curve corresponds to the standard error.

**Figure 4:**
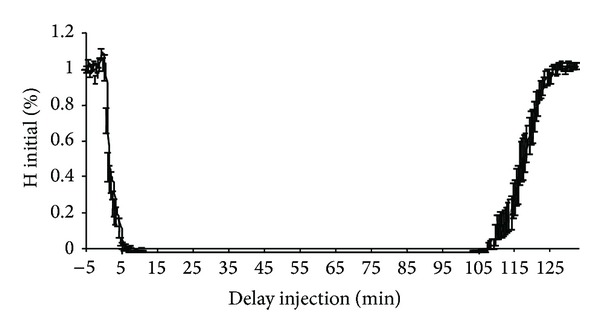
Mean time course of the amplitude of the H response expressed in relation to the baseline H response before and after application of lidocaine. Time (minutes) after application of lidocaine (performed at time 0) is shown on the *x*-axis. The mean amplitude of the H response of the 18 rats expressed in relation to the baseline H response (corresponding to the mean of 10 H responses studied before application of lidocaine) is shown on the *y*-axis. The interval shown on the curve corresponds to the standard error.

**Figure 5:**
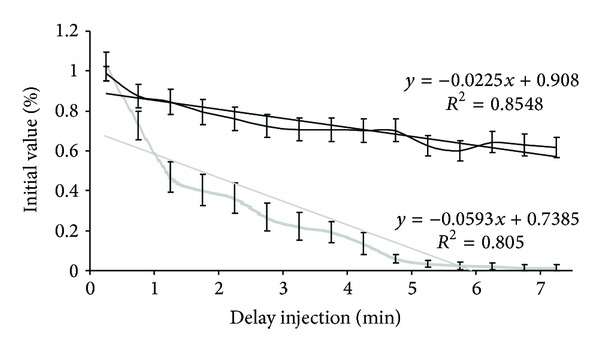
Mean time course of the amplitude of M and H responses, expressed in relation to baseline values, at the initial phase of decline after application of lidocaine. Time (minutes) after application of lidocaine (performed at time 0) is shown on the *x*-axis. The mean amplitude of the M and H responses of the 18 rats expressed in relation to baseline values (corresponding to the mean of 10 M and H responses studied before application of lidocaine) is shown on the *y*-axis. Time courses and the regression line of the M response are shown in black and the time courses and regression line of the H reflex are shown in gray. The interval shown on the curve corresponds to the standard error.

**Figure 6:**
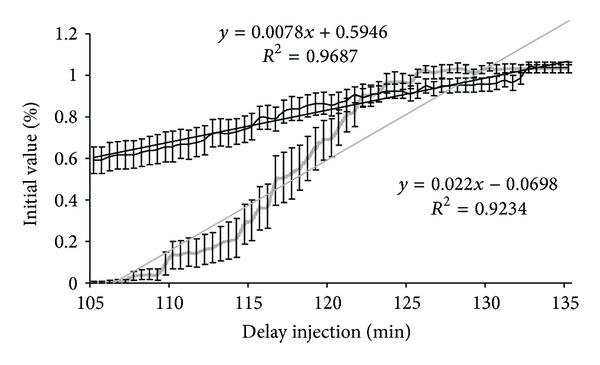
Mean time-course of the amplitude of M and H responses, expressed in relation to baseline values, during the recovery phase after application of lidocaine. Time (minutes) after application of lidocaine (performed at time 0) is shown on the *x*-axis. The mean amplitude of the M and H responses of the 18 rats expressed in relation to baseline values (corresponding to the mean of 10 M and H responses studied before application of lidocaine) is shown on the *y*-axis. Time courses and the regression line of the M response are shown in black and the time courses and regression line of the H reflex are shown in gray. The interval shown on the curve corresponds to the standard error.

All rats completed the study.  Table 1 compares the measurements performed on conscious rats and on anaesthetised rats before and after injection of lidocaine or placebo. Latencies of H and M responses were significantly increased after anaesthesia (M: latency increased by an average of 54.5 ± 8.7%, *t-*test, *P* < 0.01; H: latency increased by an average of 30.5 ± 6.2%, *t-*test, *P* < 0.01). The amplitude of H reflexes was significantly lower in anaesthetised rats (mean reduction of 18.3 ± 2.3% of the H_max⁡_/M_max⁡_ ratio, *t*-test, *P* < 0.001) while the amplitude of M responses was not significantly altered. No significant modification of M and H responses was recorded after injection of physiological saline (Table 1). No complication was observed following lidocaine injection. Figures 3 and 4 show the time courses of H and M responses expressed in relation to baseline values (mean of 10 recordings obtained before application of lidocaine) until recovery. The time course of these parameters was sufficiently similar for all animals to express the results in terms of mean values. H reflexes were abolished 5 minutes after injection and for about 100 minutes, while M responses were decreased by about one half (44.4 ± 18.7%) 7 minutes after the injection and remained at this value for about 100 minutes. More detailed analysis of the decline (Figure 5) and recovery phases (Figure 6) of H and M responses clearly demonstrated that the lidocaine action kinetic was considerably more rapid on the H reflex than on the M response: the most marked reduction of the H reflex was observed during the first minutes after the injection and was maintained with a flatter slope to reach an abolished reflex at 5 minutes. M responses decreased more slowly and the slope of their decline was significantly flatter than that of the H responses, as shown in Figure 3 (*t-*test, *P* < 0.0001). During the recovery phase (Figure 6), M responses started to return two minutes earlier than H responses, but returned more slowly to their baseline value, so that the slope of return of M responses was flatter than the slope of return of H responses. No modification of the latencies of H and M responses measured during the initial and terminal phases was observed after lidocaine injection (Table 1).

## 4. Discussion

The rat model was used to elicit H and M responses and to monitor the time course of these responses after lidocaine injection in order to more clearly define the effects of this molecule. As lidocaine injections had to be performed under anaesthesia, the first step of this study consisted of comparing M and H responses obtained in conscious rats according to the method described by Pérot and Almeida-Silveira [6] with those recorded under anaesthesia.

The amplitude and latency of H and M responses measured in conscious rats were strictly comparable to those already reported by our laboratory for age-matched rats [12, 14].

After intraperitoneal ketamine-induced anaesthesia, latencies of M and H responses increased significantly, reflecting a reduction of the conduction velocities of afferent and efferent nerve action potentials (NAP), or even those of muscle action potentials (MAP). These effects of ketamine on propagation of NAPs and MAPs have been rarely described: the cardiac action potential conduction velocity was decreased after ketamine injection [15], and Oh et al. [16], using a mouse model, recently reported a significant reduction of the conduction velocity of motor NAPs with no significant modification of the conduction velocity of sensory NAPs after combined administration of ketamine and xylazine.

Ketamine also induced a reduction of the amplitude of the H reflex response without modifying the amplitude of the direct motor M response. This result contradicts published data indicating that ketamine does not affect the H response (see Section 2), which constituted the basis for the choice of ketamine anaesthesia of the rats used in the present study. However, in their studies, Ho and Waite [13], Chiba et al. [17], and Tang and Schroeder [18] compared the effects of various anaesthetics on the H response in order to confirm that the effects of ketamine on this reflex were lower than those observed with the other anaesthetics. In other words, in contrast with our study, these previous studies did not compare the amplitude of the H reflex in conscious animals and in anaesthetised animals. Nevertheless, the H reflex is largely preserved and remains quantifiable under ketamine anaesthesia, allowing evaluation of the effects of lidocaine block on this reflex. The present study demonstrated an effect of ketamine on neuromuscular action potential conduction velocities and on the amplitude of the H reflex, although this was not the major purpose of this study.

The detailed effects of the lidocaine nerve conduction block used in clinical practice for preoperative assessment of patients with excessive spasticity of a limb segment remain poorly elucidated. The present study monitored the time course of the effects of this molecule on classical electrophysiological parameters of the myotatic reflex arc (H reflex and M response).

The latencies of the M response (or H response when a weak H response was still present) were not modified by lidocaine injection in the vicinity of the nerve, indicating the absence of effect of this molecule on MAP or sensory or motor NAP conduction velocities.

On the other hand, lidocaine induced a marked reduction of the maximum amplitude of the M response and, in the majority of cases, complete abolition of the H reflex. This more intense inhibition of reflex activity, also observed in man [2, 3], suggests preferential blockade of Ia afferent fibres compared to alpha motoneurons. Another hypothesis is that the motor block fairly selectively concerns smaller diameter alpha motoneurons, as the motoneurons, and therefore the motor units, recruited in the H reflex, are predominantly smaller diameter fibres, as reflex recruitment occurs according to the diameter principle described by Henneman and Olson [19]. In other words, if the block only affects smaller diameter motoneurons, the H reflex will be completely abolished while the M response will persist due to direct activation of the largest motoneurons. An ongoing study in our laboratory, based exclusively on afferent electroneurograms, may provide further data concerning these two hypotheses (preferential blockade of Ia afferent fibres or preferential blockade of small motoneurons).

The effect of lidocaine on the M response started 1 minute after application and reached the plateau phase after an average of 6.5 ± 1.3 minutes. The effect of lidocaine was more rapid on the H reflex (Figure 3): synchronous onset of the decline of the reflex, steeper slope of decline, and earlier plateau phase (average of 5.1 ± 0.9 minutes after application of lidocaine). This time course of the onset of the effect of lidocaine on electrophysiological responses helps to explain the clinical data reported in rats by several authors [7–11]. According to these authors, the effects of lidocaine are observed 1 to 10 minutes after injection and primarily affect proprioceptive sensitivity (Ia or A alpha afferent fibres), followed by motor function (*α* motoneurons) and finally superficial then deep cutaneous nociception (C and A delta afferent fibres) [7, 9]. The more rapid action on the H reflex (Figure 5) is concordant with these clinical findings. The study of the onset phase of the lidocaine block therefore demonstrates a more rapid and more intense action of this molecule on Ia fibres. Lidocaine is therefore more effective in that it acts on large diameter fibres, which would tend to be in favour of the first hypothesis formulated earlier: preferential blockade of Ia afferent fibres.

The recovery phase began more than 100 minutes after application of lidocaine for the M response with return to baseline amplitude after 132 minutes. Return of the H response was observed slightly later but was just as rapid as restoration of the M response. Clinical data are concordant with this recovery kinetic: return of nociception then motor function and finally proprioception, with complete recovery of all modalities between 70 and 120 minutes after lidocaine injection [7–11]. It is classically reported in man that the effect of lidocaine block lasts between 2 and 4 hours depending on the anatomical site [20].

These differences in the duration of action of lidocaine may be due to the fact that our study was based on electrophysiological parameters, while previous studies were based on clinical parameters [7–10], as it is obviously easier to demonstrate a difference in terms of the amplitude of EMG responses than on subjective clinical tests. Furthermore, the afferent fibres assessed by the H reflex obviously do not participate in nociception, which is the first modality to recover after lidocaine block [7].

## 5. Conclusion

Ketamine, an anaesthetic reputed not to modify reflex responses, clearly induces a slight reduction of reflex response and increases the latency of direct motor M and H reflex responses by slowing conduction velocities.

This electromyographic assessment of H reflexes and direct motor M responses in animals confirms data previously reported in man on the effect of lidocaine on peripheral nerves to block the myotatic reflex arc, which is exacerbated in spastic subjects. Lidocaine therefore has a more intense and more rapid action on large diameter Ia proprioceptive fibres, although it may also have a more intense action on smaller diameter motoneurons. The differential effect of lidocaine on nerve fibres could therefore be diameter dependent. This preferential effect of lidocaine on larger diameter afferent fibres could be confirmed by studying afferent electroneurograms after lidocaine injection.

## Figures and Tables

**Table 1:** Mean (±standard deviation) of latencies and amplitudes of M responses and H responses expressed in relation to the maximum M response, measured in prone conscious rats and then rats anaesthetised by intraperitoneal ketamine and placed in the lateral supine position before (pre) and after (post) lidocaine injection during the plateau phase. Finally, the two right-hand columns indicate the values recorded before (pre) and after (post) injection of placebo under the same conditions as those used for lidocaine injection. The post-lidocaine latency of the H reflex corresponds to the mean latency of the H reflexes persisting on several paws after lidocaine injection. The “pre-lidocaine” column shows statistical comparison of the values obtained in “conscious rats” and “pre-lidocaine”, while the “post-lidocaine” column shows statistical comparison of  “pre-lidocaine” and “post-lidocaine” values. No statistically significant difference was observed between “preplacebo” and “postplacebo” values.

	Conscious rats (*n* = 22)	Anaesthetised rats (*n* = 22)			
		Lidocaine (*n* = 18)		Placebo (*n* = 4)	
		Pre	Post	Pre	Post
Latency (ms)					
M response	1.1 ± 0.1	1.7 ± 0.5**	1.7 ± 0.6	1.7 ± 0.3	1.7 ± 0.4
H reflex	5.9 ± 0.4	7.7 ± 1.1**	7.7 ± 1.0	7.7 ± 0.8	7.7 ± 0.9
Amplitude of M response (mV)	39.2 ± 7.5	35.4 ± 6.6	19.1 ± 6.4***	35.3 ± 3.2	35.4 ± 3.9
H_max_/M_max_ (%)	47.1 ± 15.1	38.5 ± 10.8***	0.0 ± 0.2***	38.2 ± 4.3	38.1 ± 3.9

**Highly significant difference (*t*-test; *P* < 0.01). ***Very highly significant difference (*t*-test; *P* < 0.001).

## References

[1] Tardieu G, Harriga J (1964). Traitement des raideurs musculaires d'origine centrale par infiltration d'alcool dilué (résultats de 500 injections). *Archives Françaises de Pédiatrie*.

[2] Buffenoir K, Decq P, Lefaucheur J-P (2005). Interest of peripheral anesthetic blocks as a diagnosis and prognosis tool in patients with spastic equinus foot: a clinical and electrophysiological study of the effects of block of nerve branches to the triceps surae muscle. *Clinical Neurophysiology*.

[3] Deltombe T, Jamart J, Hanson P, Gustin T (2008). Soleus H reflex and motor unit number estimation after tibial nerve block and neurotomy in patients with spastic equinus foot. *Neurophysiologie Clinique*.

[4] Bleyenheuft C, Detrembleur C, Deltombe T, Fomekong E, Lejeune TM (2008). Quantitative assessment of anaesthetic nerve block and neurotomy in spastic equinus foot: a review of two cases. *Journal of Rehabilitation Medicine*.

[5] Filipetti P, Decq P (2003). L'apport des blocs anesthésiques dans l'évaluation du patient spastique. A propos d'une série de 815 blocs moteurs. *Neurochirurgie*.

[6] Pérot C, Almeida-Silveira MI (1994). The human H and T reflex methodologies applied to the rat. *Journal of Neuroscience Methods*.

[7] Nakamura T, Popitz-Bergez F, Birknes J, Strichartz GR (2003). The critical role of concentration for lidocaine block of peripheral nerve in vivo. Studies of function and drug uptake in the rat. *Anesthesiology*.

[8] Huang JH, Thalhammer JG, Raymond SA, Strichartz GR (1997). Susceptibility to lidocaine of impulses in different somatosensory afferent fibers of rat sciatic nerve. *Journal of Pharmacology and Experimental Therapeutics*.

[9] Popitz-Bergez FA, Leeson S, Strichartz GR, Thalhammer JG (1995). Relation between functional deficit and intraneural local anesthetic during peripheral nerve block: a study in the rat sciatic nerve. *Anesthesiology*.

[10] Thalhammer JG, Vladimirova M, Bershadsky B, Strichartz GR (1995). Neurologic evaluation of the rat during sciatic nerve block with lidocaine. *Anesthesiology*.

[11] Fink BR, Aasheim G, Kish SJ, Croley TS (1975). Neurokinetics of lidocaine in the infraorbital nerve of the rat in vivo: relation to sensory block. *Anesthesiology*.

[12] Anderson J, Almeida-Silveira MI, Pérot C (1999). Reflex and muscular adaptations in rat soleus muscle after hindlimb suspension. *Journal of Experimental Biology*.

[13] Ho SM, Waite PME (2002). Effects of different anesthetics on the paired-pulse depression of the H reflex in adult rat. *Experimental Neurology*.

[14] Pérot C, Gamet D, Canon F (2008). Evolution of reflex excitability all along the life of Wistar rats. 75ème congrès annuel de la Société de Physiologie. *Fundamental & Clinical Pharmacology*.

[15] Hara Y, Tamagawa M, Nakaya H (1994). The effects of ketamine on conduction velocity and maximum rate of rise of action potential upstroke in guinea pig papillary muscles: comparison with quinidine. *Anesthesia and Analgesia*.

[16] Oh SS, Hayes JM, Sims-Robinson C, Sullivan KA, Feldman EL (2010). The effects of anesthesia on measures of nerve conduction velocity in male C57Bl6/J mice. *Neuroscience Letters*.

[17] Chiba A, Nakanishi H, Hiruma S, Satou T, Hashimoto S, Chichibu S (1998). Magnetically induced motor evoked potentials and H-reflex during nembutal and ketamine anesthesia administration in rats. *Research Communications in Molecular Pathology and Pharmacology*.

[18] Tang AH, Schroeder LA (1973). Spinal cord depressant effects of ketamine and etoxadrol in the cat and the rat. *Anesthesiology*.

[19] Henneman E, Olson CB (1965). Relations between structure and function in the design of skeletal muscles. *Journal of Neurophysiology*.

[20] O’donnell BD, Iohom G (2009). An estimation of the minimum effective anesthetic volume of 2% lidocaine in ultrasound-guided axillary brachial plexus block. *Anesthesiology*.

